# Perceiving pain alters body perception: the effects of acute pain on body image and sensory testing

**DOI:** 10.1097/j.pain.0000000000003872

**Published:** 2025-11-26

**Authors:** Aleksandra Budzisz, Wacław M. Adamczyk, Kerstin Luedtke

**Affiliations:** a Pain Research Group, Institute of Psychology, Jagiellonian University, Kraków, Poland; b Laboratory of Pain Research, Institute of Physiotherapy and Health Sciences, The Jerzy Kukuczka Academy of Physical Education, Katowice, Poland; c Department of Physiotherapy, Pain and Exercise Research Luebeck (P.E.R.L), Institute of Health Sciences, Universität zu Lübeck, Luebeck, Germany

**Keywords:** Body image, Pain, Acute pain, Distorted body image

## Abstract

This study investigated the effects of experimentally induced acute pain on body image and sensory testing. A total of 91 pain-free participants were randomly assigned to 1 of 3 groups: injection (pain), sham injection, or control. Measures were taken 3 times in each group: at baseline, during the testing phase, and once the pain had subsided. The study included both sensory testing and body image measures. Participants in the injection group showed significant increases in body image distortion during the testing phase (*P* < 0.001, η^2^ = 0.19), alongside larger area of pain distribution (*P* < 0.001, η^2^ = 0.40) and greater size distortion of the lower back (*P* < 0.001, η^2^ = 0.14) compared with the other groups. A drawing task further revealed that the painful body part was perceived as larger (*P* = 0.01, η^2^ = 0.14), particularly on the right side. Body esteem indicated a subtle but significant decrease in body satisfaction during pain (*P* = 0.05, η^2^ = 0.06). Sensory testing measures remained unchanged, suggesting intact sensory processing. Correlation analyses showed positive associations between Polish version of the Fremantle Back Awareness Questionnaire scores and perceived pain area, pain intensity, pain catastrophizing, and general fear. These findings highlight the selective influence of acute pain on body image, suggesting that perceptual and emotional factors—driven by higher-order mechanisms play a central role in pain-related body distortions rather than changes in basic sensory processing.

## Abstract 2

Supplemental Digital Content is Available in the Text.

Acute pain can distort body image, suggesting that distorted body image is more closely related to perceptual and emotional factors than to sensory changes.

## 1. Introduction

Body image is a multidimensional construct encompassing perceptual, behavioural, emotional, and attitudinal components. These aspects shape how individuals experience and interact with their bodies across the lifespan.^8,9,18^ It includes attitudinal dimensions (such as attitudes towards the body), as well as perceptual and behavioural aspects related to body size estimation and body-related actions.^9,25,40^ Although body image, body awareness, and interoception may present similar symptoms in certain cases, they originate from different theoretical concepts.^34^

The way in which the body is perceived depends not only on internal factors, such as emotions, attitudes, or perceptual processing, but also on bodily signals, such as pain. Experiencing pain has been associated with a distorted body image and higher levels of body dissatisfaction,^6,41^ suggesting that pain is often accompanied by a more negative perception of the body. In contrast, individuals who do not experience pain in their daily lives, tend to report more positive body attitudes, which have been linked to less pain sensitivity (higher pain thresholds).^44^

Focusing on differences in body image as a function of pain type (acute vs chronic), research shows that individuals with chronic pain tend to exhibit a more negative body image compared with those experiencing acute pain.^23^ In studies where individuals with chronic pain were asked to visually represent their own bodies—by outlining their perceived body shape—painful body parts were perceived as swollen, shrunken, or difficult to outline.^31,33^ In addition, in this population, perceiving the painful body part as larger (swollen) has been associated with decreased tactile discrimination.^33^

The perceptual component of body image (as observed during pain) encompasses alterations in perceived size and shape, sense of ownership, and proprioceptive awareness—particularly in individuals with chronic pain.^46^ A recent systematic review and meta-analysis provided further insight into body image distortions within this population, identifying that individuals with chronic pain exhibited greater body image distortion than pain-free individuals.^6^ Furthermore, higher pain intensity was associated with more pronounced perceptual and attitudinal disturbances, whereas pain duration had no significant effect.^6^

Despite the importance of above-mentioned findings, the causal relationship between pain and body image remains unclear. Most existing studies are correlational, leaving open the question of whether pain itself induces changes in body image, or whether both phenomena arise from shared underlying mechanisms such as emotional distress, attentional bias, or potentially cortical reorganization. To address this gap, an experimental study was conducted in which pain was temporarily induced and immediate changes in body image were assessed to provide evidence for a causal influence of pain on body image and point out characteristics of these changes.

## 2. Methods

Study procedures were preregistered at https://doi.org/10.17605/OSF.IO/VHXTG. The protocol of the study was approved by the local Bioethics Committee (No. 3/2022). The study was conducted at the Academy of Physical Education in Katowice, strictly following the recommendations of the Declaration of Helsinki.^50^ The data collection was carried out in the ISO-certified Laboratory of Pain Research.

### 2.1. Participants

Ninety-one pain-free individuals were recruited for participation in this study. The advertisement for this study was distributed by social media, institutional mailing lists, and printed flyers. Participants were compensated with 90 ZŁ for their participation. Participants were familiarized with the study's objectives before entering the laboratory. Upon arrival at the laboratory, they provided written informed consent and were explicitly informed that they could withdraw their consent at any point during the experiment without providing a reason.

The inclusion criteria were as follows: (1) age over 18 years and (2) sufficient cognitive and language ability to understand and follow oral and written instructions. The exclusion criteria were as follows: (1) experiencing pain at the time of examination, (2) experiencing pain lasting more than 1 hour within the last 24 hours, (3) experiencing pain in the last 7 days, (4) intake of any analgesic medication, (5) intake of alcohol or psychoactive substances within 24 hours before participation, (6) any diagnosis of health issues (musculoskeletal, neurological, cardiological, psychiatric, or other), (7) allergy to hypertonic saline, (8) pregnancy, (9) diagnosed postural misalignment, (10) previous spine surgery, and (11) spinal implants.

### 2.2. Sample size

The sample size was determined based on an á priori power analysis (2-tailed *t* test), using G*Power,^15^ to detect a difference between one of the control groups and experimental group in Polish version of the Fremantle Back Awareness Questionnaire score (FreBAQ_PL). This comparison was selected as the most conservative and theoretically relevant contrast. The analysis indicated that a total of N = 90 participants (n = 30 per group) would be required to detect a large effect size (Cohen *d* = 0.8),^10^ with α = 0.05 and 80% power.

Although the primary hypothesis was tested using a repeated-measures analysis of variance (ANOVA), the *t*-test-based estimate served as a cautious lower bound. For completeness, a post hoc power analysis using a 1-way ANOVA (fixed effects, omnibus test) with *f* = 0.4, α = 0.05, and power = 0.80 indicated that a minimum of 66 participants (n = 22 per group) would suffice. Thus, the final sample of 90 provides sufficient power for both the planned comparisons and the overall ANOVA framework while allowing for potential dropout and increasing the robustness of the findings.

The decision to use an effect size of *d* = 0.8 was based on findings from a recent systematic review and meta-analysis,^6^ which reported a pooled effect size of 1.33 for FreBAQ differences between pain and nonpain groups. Given that our study induced pain experimentally in a healthy population—likely leading to smaller effects—we selected a more conservative yet still substantial estimate in line with the expected magnitude of perceptual changes under acute, nonclinical conditions. This approach is consistent with recommendations to balance practical constraints and informational value in empirical research.^22^

### 2.3. Study design and types of interventions

**Figure 1.**
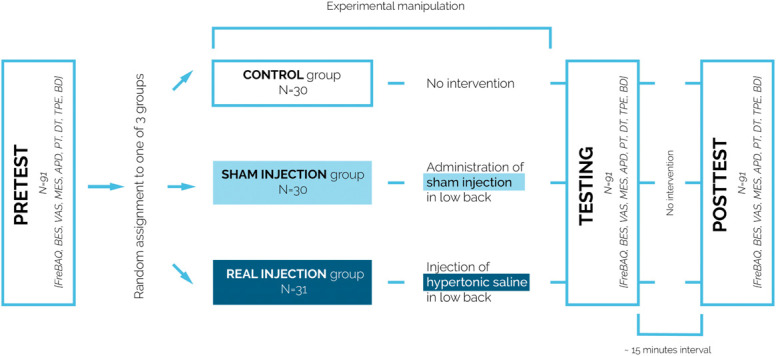
Study design. The randomization process conducted before the experiment determined group allocation, the order of applied tests for each assessment, and the assessment site (left/right). During the pretest, participants completed questionnaires (FreBAQ_PL, BES, VAS, MES) and performed the tasks (APD, PT, DT, TPE, BD). After the pretest, the physician carried out the experimental manipulation (injection, sham injection) or no intervention (control group). Immediately after the manipulation, the second assessment (testing phase) was conducted. The posttest took place once the pain had subsided or approximately 15 minutes after testing. APD, area of pain distribution; BES, Body Esteem Scale; BD, Body drawings; DT, detection threshold; FreBAQ_PL, Polish version of the Fremantle Back Awareness Questionnaire; MES, Magnitude Estimation Scale; PT, pain threshold; TPE, two-point estimation task; VAS, visual analogue scale.

**Figure 2.**
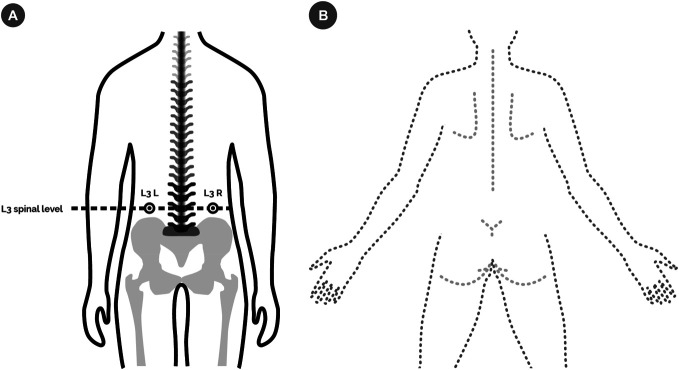
Study details. (A) Determination of measurement points. A point was marked on the skin surface 5 cm laterally from the spinal process of L3, on the left or right side. This point indicated the testing location and, if applicable, the site for injection or sham injection. The marked point was used for PT, DT, and TPE assessments, as well as for experimental manipulation (injection or sham injection). (B) The body drawing task was performed on an iPad. Participants were presented with a digital outline and instructed to add lines representing how they perceived the shape, size, and symmetry of their back. The instruction provided to participants was as follows: “*Using the tablet, draw the outline of your back and the shape of your spine as you imagine it in your mind. Focus on how the right and left sides of your body feel in relation to each other. Remember, this drawing should reflect how your back feels to you, not how you think it looks. Pay close attention to the sensations in each area of your back while you draw. Let your perception guide your drawing*.” This instruction was adapted from previous studies using the back drawing task.^31,33^ DT, detection threshold; PT, pain threshold; TPE, two-point estimation task.

The study was based on within- and between-subjects design. Pain-free individuals were randomly assigned to 1 of 3 groups: injection, sham injection, and control (Fig. 1). They were informed that they can be allocated to a control or an injection group (thereby hiding the possibility of a sham injection). After obtaining written consent, participants were asked to lie down in a prone position with their head placed in the designated position on the couch. The physician palpated the L3 spinous process and marked the injection site 50 mm to the right or left of the spinal process^3^ (Fig. 2A).

During the experiment, 2 investigators were present: a physician and the assessor (A.B.). The physician was responsible for performing the experimental interventions (injection, sham injection, no intervention), in the absence of the assessor. The assessor collected the behavioural data. Randomization was performed using a PHP (v7.3) script and a pseudorandom number generator with finite numbers of cases. Randomization included the group allocation (injection, sham injection, control), the side of stimulation (left, right) and the order of applied tests. The assessor remained blinded of the participants' group assignments throughout the entire procedure and was no present in the laboratory during the experimental interventions (injection, sham injection, control). To maintain effective blinding across all experimental conditions, postintervention procedures were standardized regardless of group allocation. Specifically, a small adhesive plaster was applied to the marked injection site on the lower back of each participant (including those in the injection, sham and control groups). This approach was feasible because the assessor—responsible for all measurements and interactions with participants—was blinded to group allocation and was not present during the intervention phase. Participants were also not informed about the expected duration, intensity, or qualitative characteristics of postinjection pain, thereby minimizing the likelihood that subjective pain experience could inform group assignment. To further evaluate the credibility of the blinding, all participants were asked at the end of the study whether they believed they had an injection during the study.

In the injection group, acute pain was induced by administering a 0.7% hypertonic saline solution into the lumbar longissimus dorsi muscle. The injection was performed using a 0.8 × 40-mm needle and a 5-mL syringe (1 mL was injected), targeting a site 50 mm lateral to the L3 spinous process at a depth of 30 mm.^3^ Each injection was conducted under portable ultrasound imaging with a 7- to 11-MHz linear probe and a 2.5- to 5-MHz convex probe (Sonoscape E2; Sonoscape, Shenzhen, China) guidance to ensure that the stimuli were applied at the same depth, area, and tissue for each participant.

The sham group was implemented to simulate the context and sensory characteristics of an injection, without inducing lasting pain. The sham injection mimicked the injection procedure including ultrasound imaging but without piercing the skin. The sensation of an injection was simulated using a weighted stimulus (flat contact area, 0.25 mm in diameter; PinPrick; MRC Systems GmbH, Heidelberg, Germany). A stimulus of 512 mN was chosen because it has been shown to activate cutaneous nociceptors^20^ and was used successfully in previous studies.^2,3^ This single stimulation of nociceptors was used to create a convincing sensation of injection, serving as the nocebo-like pain condition for the sham group.

In the control group, no intervention between measures was performed. Participants were asked to wait in their prone position for the same duration of time as those in the intervention groups.

In the course of the study, a set of assessments was administered to each group 3 times. The first set of assessments was conducted before the intervention (pretest). The second set of assessments was conducted during the pain/sham/waiting phase (ie, the testing phase). The third set of assessments (posttest) was conducted after the pain had subsided or 15 minutes after the intervention in the sham and control groups.

### 2.4. Measurements

#### 2.4.1. Primary outcome

To quantify body image distortion, the primary outcome measure was the FreBAQ_PL, which assesses the perceptual dimension of body image distortion.^7,46^ It is a 9-item tool that captures the impaired perception of the lower back because of perceived pain and its effect on the body image. The score of the questionnaire is 0 to 36 and is calculated as a sum of responses (Likert scales 0-4). Higher results indicate higher body image distortion.

#### 2.4.2. Secondary outcomes

The intensity of the experimentally induced pain (PI) was rated on a visual analog scale (0-100 mm). Participants (in each group) were asked for the perceived pain intensity (PI) every 60 seconds, beginning immediately after the intervention (injection, sham injection) until the pain had subsided.

The area of pain distribution (APD) was assessed by using a digital software for Pain Drawing Analysis^21^ to calculate the percentage score of body areas perceived as painful. Participants were asked to mark the area of the body where they were currently experiencing pain.

Sensory testing included electrical detection threshold (DT),^37^ electrical pain threshold (PT),^35^ and the 2-point estimation task (TPE).^32^ Detection threshold was assessed using electrical stimulation on the previously marked point at the lower back by using Digitimer DS-7A constant current high voltage stimulator (Digitimer Ltd, Hertfordshire, England). The current of the stimuli started at 0 mA with increments of 0.5 mA, until the participant reported stimulus perception. The intensity of the stimuli reported as perceived was documented as the tactile threshold.^37^ Pain thresholds were determined in a similar way; however, participants were asked to report the first sensation of pain. The intensity of the stimuli first reported as painful was taken as the value of pain threshold.^35^ The TPE test was used as a feasible alternative to 2-point discrimination.^32^ During this test, 2 von Frey filaments (300 g/6.65) (Bioseb, Vitrolles, France) touched the surface of the skin of the back simultaneously with the predetermined point exactly in the middle of the filaments. Tactile stimuli with a constant distance (120 mm) were applied along (vertical) or across (horizontal) the spine. Immediately after stimulus application, the subject was asked to estimate, using a second caliper (held in his/her hands), the distance between the applied stimuli. The numerical value (invisible to the subject) of his/her estimated distance was recorded. The vertical and horizontal measurement was repeated twice.

Three-dimensional body image perception was assessed through a finger size estimation task.^43^ Participants estimated the size of their index finger without visual or tactile reference by determining whether their finger would fit into a series of metal ring gauges (Kociuba, Kraków, Poland). The set included 10 rings, each 3.6 mm thick, with inner diameters ranging from 10 to 25 mm (European sizes 6-33 mm).

The Magnitude Estimation Scale (MES) was used to estimate the perceptual distortion of the lower back—the part of the body, where experimental pain was induced. Participants were asked to indicate whether the affected area appeared smaller or larger compared with an unaffected area. This allowed the assessment of changes in the subjective perception of the size of the lower back following the induction of pain.^12,13^ The MES ranges from −100 to +100, where −100 indicates a maximal perceived decrease in size, 0 represents no change, and +100 reflects a maximal perceived increase in the size of the painful region.

To capture the perceived shape and size of the back, the Body Drawing (BD) task was performed. In this task, participants drew the shape of their backs on an iPad (iPad 9.7; Apple Inc, Cork, Republic of Ireland) (Fig. 2B).

Body esteem was assessed, using the Body Esteem Scale (BES). It consists of 35 items enabling to capture the attitude towards the body.^18,24^ Responses were given on a 5-point Likert scale (1 = strong negative feelings, 5 = strong positive feelings). The sum was calculated for all items; higher scores indicate more positive attitudes towards the body.

This comprehensive set of outcomes was selected to reflect the multidimensional nature of body image, capturing its perceptual (eg, perceptual distortion of the back, APD), perceptual with cognitive-affective elements (eg, FreBAQ_PL), behavioral (eg, finger size estimation, body drawing), and attitudinal (eg, BES) components. Behavioral tasks such as the finger size estimation and body drawing allowed to probe perceived body size and shape without relying solely on verbal self-report. By incorporating both region-specific measures (eg, back-related distortions) and more generalized representations (eg, finger estimation), the spatial specificity of pain-induced changes was explored. Sensory testing (DT, PT, TPE) served as a reference point to evaluate whether body image distortions were accompanied by alterations in sensory processing or whether such changes reflected higher-level perceptual reorganization independent of peripheral sensory function.

### 2.5. Descriptive data

Before the experimental procedures, participants completed general assessments using visual analogue scales (VAS) to assess their psychological predispositions to pain-related experiences. These assessments included ratings of general fear of pain, where participants indicated their general fear of pain (GF) and fear of injections, using a continuous scale with anchors labelled “*not fearful at all*” and “*very fearful*.” Participants also provided a measure of pain expectancy, rating the anticipated intensity of pain from the injection, (prior to the procedure) using a scale with anchors ranging from “*no pain*” to “*most intense pain*.” After the experimental intervention, VAS was used to assess current pain intensity, with the same scale anchors of “*no pain*” and “*most intense pain*.”

At the end of the experiment, demographic data including sex assigned at birth, gender identity, age, height, and actual and desired weight were also recorded, and the difference between them was used to calculate the Weight Discrepancy Index (WDI).

In addition, at the end of the experiment, participants completed the Pain Catastrophizing Scale, a tool that assesses the extent to which individuals magnify, ruminate on, and feel helpless about their pain.^38,39^

### 2.6. Statistical analysis

The preparatory steps before statistical analysis included converting the VAS scores into numerical values, similarly to all outcomes (ie, transforming responses into numerical scores). For the back drawing task, the area was calculated by summing the number of pixels within the drawn shape. This area was further calculated separately for the left and right sides, depending on the side on which the assessment (and intervention) was performed.

Baseline differences in descriptive statistics were evaluated using a 1-way ANOVA with Bonferroni correction, where “*group*” served as the between-subject factor.

Repeated-measures ANOVA was used with “*group*” (experimental, sham injection and control) as the between-subject factor and “*assessment*” (assessment 1, assessment 2, and assessment 3) as the within-subject factor. The analysis was followed by planned Bonferroni-corrected comparisons.

The relationship between variables was assessed using Pearson's correlation test, to explore the relationship between reported pain intensity and all other assessed variables. To account for the risk of type I error because of multiple comparisons, a Bonferroni correction was applied to the correlational analysis involving FreBAQ_PL scores and 6 outcome variables, resulting in a corrected alpha level of 0.0083.

The statistical analyses were performed using IBM Statistical Package for Social Science (SPSS Version 28, Armonk, NY). The level of significance was set at *P* < 0.05.

## 3. Results

Ninety-one participants (45% female) were included in the data analyses (Supplementary File, Table 1, http://links.lww.com/PAIN/C420). All participants reported their gender identity as consistent with their sex assigned at birth (M/F). The mean age was 22.67 years (SD 3.44), the mean height was 177.93 cm (SD 31.08), and the mean weight was 72,15 kg (SD 12.71), resulting in a mean body mass index of 23.36 kg/m^2^ (SD 3.86). The results showed that 97% of those in the injection group, and 73% of those in the sham group, reported believing they had received an injection. In contrast, none of the control group participants endorsed this belief (Supplementary File, Table 1, http://links.lww.com/PAIN/C420).

**Figure 3.**
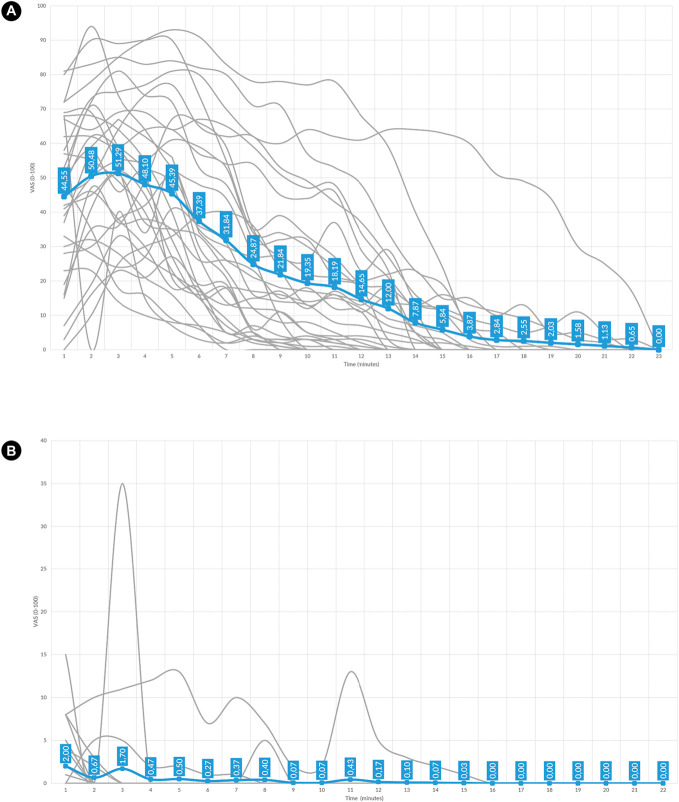
Graphical representation of reported pain intensity during testing phase. (A) Pain intensity in injection group. (B) Pain intensity in sham injection group. Gray lines represent individual pain intensity trajectories. Bold blue line presents an average curve from the group. The blue labels indicate the mean pain intensity for each minute of the testing phase.

Pain intensity ratings differed significantly across the 3 experimental conditions. Participants in the injection group reported the highest mean pain intensity (M = 20.38, SD = 13.53) (Fig. 3A), which was significantly greater than both the sham group (M = 0.33, SD = 0.95; *P* < 0.001) (Fig. 3B) and the control group (M = 0.00, SD = 0.00; *P* < 0.001). Mild to high pain intensity in the injection group was observed, and the pain lasted over 20 minutes, whereas in the sham group, only a very a brief, low-level sensation pain was reported.

**Figure 4.**
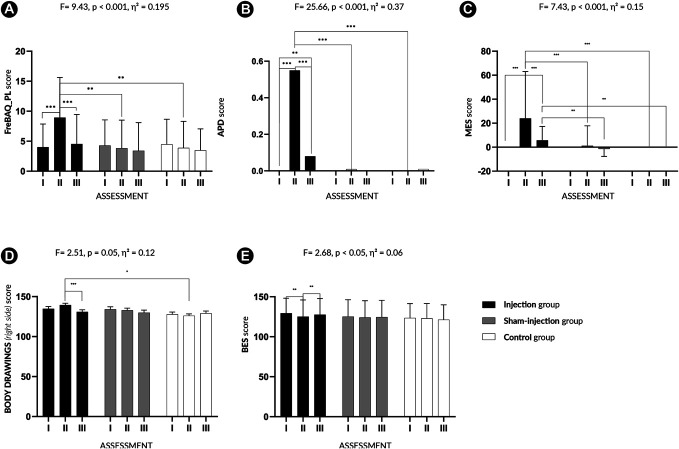
Graphical representation of the main effects obtained in the study. Colours represent different groups. Assessment I (pretest), II (testing phase), III (posttest). (A) Fremantle Back Awareness Questionnaire. (B) Area of pain distortion. (C) Magnitude Estimation Scale. (D) Body drawings of the right side. (E) Body Esteem Scale. ***P < 0.001, **P = 0.01 to 0.04, *P < 0.05.

A repeated-measures ANOVA with Greenhouse–Geisser correction revealed a statistically significant 2-way interaction between “group” and “assessment” on body image distortion, as measured by the FreBAQ_PL, with a large effect size, F(3.30, 145.03) = 9.99, *P* < 0.001, η^2^ = 0.185 (Supplementary File, Table 2, http://links.lww.com/PAIN/C420). After including GF and WDI as covariates, the interaction effect remained significant and slightly increased, F(3.33, 143.32) = 9.43, *P* < 0.001, η^2^ = 0.195 (Fig. 4A, Supplementary File, Table 2a, http://links.lww.com/PAIN/C420). However, the effect of assessment became nonsignificant (F[1,66, 143,32] = 0.21, *P* = 0.768), suggesting that changes in FreBAQ_PL over time were partly explained by baseline differences in GF and WDI. Pairwise comparisons with Bonferroni correction showed that during the acute pain (testing) phase, the injection group had significantly higher FreBAQ_PL scores (M = 8.94, SD = 6.69) compared with the sham injection (M = 3.83, SD = 4.67) and control groups (M = 3.87, SD = 4.44) (*P* < 0.01), indicating greater body image distortion associated with induced pain. Within-group analyses revealed no significant changes in body image distortion over time (after adding covariates) (F[1.66, 143.32] = 9.43, *P* = 0.16, η^2^ = 0.042). Within-group pairwise comparisons confirmed that body image distortion significantly increased only in the injection group (*P* < 0.001) from pretest (M = 4.00, SD = 3.86) to the testing phase (M = 8.94, SD = 6.69) and decreased in the posttest (M = 4.52, SD = 4.94). No such changes were observed in the sham injection or control groups, where FreBAQ_PL scores remained stable across assessments (Supplementary File, Tables 2b, 2c, http://links.lww.com/PAIN/C420).

No significant main effect of interaction (assessment and group) was found for pain thresholds (F[2.61, 115.19] = 0.21, *P* = 0.87, η^2^ = 0.005), detection thresholds (F[3.47, 152.70] = 0.70, *P* = 0.57, η^2^ = 0.016), or TPE, assessed vertically (F[3.13, 62.65] = 1.40, *P* = 0.25, η^2^ = 0.06) or horizontally (F[3.72, 74.34] = 0.09, *P* = 0.98, η^2^ < 0.01), and for the finger size estimation task (F[2.92, 176.00] = 0.68, *P* = 0.68, η^2^ = 0.02). Including covariates did not alter the pattern of significant interaction effects, although slight changes in effect size were observed (Supplementary File, Tables 3–7, http://links.lww.com/PAIN/C420).

A significant main effect of interaction between assessment and group with a large effect size was found (with included GF and WDI as covariates) for APD scores (F[3.03, 130.28] = 25.66, *P* < 0.001, η^2^ = 0.37) (Fig. 4B, Supplementary File, Tables 8, 8a, http://links.lww.com/PAIN/C420).

The injection group reported a significantly larger perceived pain area during the testing phase (*P* < 0.01), as indicated by pairwise comparisons (M = 0.55%, SD = 0.00%) compared with the sham injection (M = 0.01%, SD = 0.00%) and control groups (M = 0.04%, SD = 0.00%). Within-group analyses (with covariates) indicated a significant increase in APD over time in the assessments (F[1.52, 130.28] = 3.48, *P* = 0.046, η^2^ = 0.039). The observed difference occurred only in the injection group, with a significant increase from pretest (M = 0.00%, SD = 0.00%) to the testing phase (M = 0.55%, SD = 0.00%), *P* < 0.001, followed by a return to baseline in the post-test (M = 0.08%, SD = 0.00%) *P* < 0.001. No significant changes were observed in the sham or control groups over time (Supplementary File, Tables 8b, 8c, http://links.lww.com/PAIN/C420).

The perceptual distortion of the lower back score also showed an interaction between assessment and group (with WDI and GF covariates), with a large effect size (F [2.31, 99.49] = 7.43, *P* < 0.001, η^2^ = 0.15) indicating notable variations in perceived body size during acute pain (Fig. 4C, Supplementary File, Tables 9, 9a, http://links.lww.com/PAIN/C420). Pairwise comparisons (*P* < 0.001) revealed that in the testing phase, the injection group demonstrated the highest perceived body size distortion (M = 23.96, SD = 39.06) compared with the sham injection (M = 1.10, SD = 16.68) and control groups (M = 0.00, SD = 0.00). Within-group comparisons with covariates revealed that scores did not change over time (F[1.16, 99.49] = 0.16, *P* = 0.73, η^2^ = 0.002). Pairwise comparisons revealed, that in the injection group, the score of perceptual distortion of the lower back score significantly increased from pretest (M = 0.00, SD = 0.00) to the testing phase (M = 23.96, SD = 39.06) (*P* < 0.001), followed by a significant decrease in the posttest (M = 5.74, SD = 11.55) (*P* < 0.001), as well as between pretest and posttest (*P* < 0.001). No significant changes were observed in the sham or control groups (Supplementary File, Tables 8b, 8c, http://links.lww.com/PAIN/C420).

In the body drawing task, a moderate significant interaction effect (assessment × group) was found for the perceived size of the right side of the back, specifically when stimulation and assessment were both conducted on the right side and with GF and WDI included as covariates (F[3.88, 73.76] = 2.51, *P* = 0.05, η^2^ = 0.12) (Fig. 4D, Supplementary File, Tables 10, 10a, http://links.lww.com/PAIN/C420). The injection group perceived a larger back area in the testing phase (M = 139.41, SD = 2.26) compared with the sham injection (M = 132.88, SD = 2.69) and control group (M = 126.00, SD = 2.49), *P* < 0.001. Within-group analysis showed no significant interaction over time in the performed assessments (F[1.96, 78.34] = 2.22, *P* = 0.11, η^2^ = 0.05). In the injection group, a significant decrease in the size of drawn body shapes was observed from the testing phase (M = 139.41, SD = 2.26) to the posttest phase (M = 130.93, SD = 2.60) (*P* < 0.001). No significant changes were observed in the sham or control groups (Supplementary File, Tables 10b, 10c, http://links.lww.com/PAIN/C420). In the body drawing task involving the left side of the body and its assessment, no significant main effect or interaction was observed (F[3.49, 75.05] = 0.66, *P* = 0.60, η^2^ = 0.03) (Supplementary File, Table 11, 11a, http://links.lww.com/PAIN/C420).

Results of BES (with covariates) indicated a small but meaningful interaction effect (assessment × group), suggesting differences in self-reported body esteem (F[3.51, 150.79] = 2.68, *P* < 0.05, η^2^ = 0.06) (Fig. 4E, Supplementary File, Tables 12, 12a, http://links.lww.com/PAIN/C420). Within-group analyses did not reveal a significant change over time in BES scores (F[1.75, 150.79] = 1.73, *P* = 0.19, η^2^ = 0.02) (Supplementary File, Table 12b, http://links.lww.com/PAIN/C420). Within-group pairwise comparisons indicated a significant decrease in BES scores in the injection group (*P* < 0.001) from pretest (M = 129.48, SD = 18.67) to the testing phase (M = 125.23, SD = 20.82), followed by partial recovery in the posttest, *P* = 0.12 (M = 127.65, SD = 19.99) (Supplementary File, Table 12c, http://links.lww.com/PAIN/C420). No significant within-group changes were observed in the sham or control groups.

The correlational analyses were performed on FreBAQ_PL results from the testing phase (except for fear variables, which were assessed before pretest) and included participants from all groups. FreBAQ_PL scores were moderately positively correlated with APD (r = 0.54, *P* < 0.001), indicating that greater body image distortion was associated with a larger perceived pain area. Additional moderate positive correlations were also observed with perceptual distortion of the lower back (*r* = 0.38, *P* < 0.001), experienced pain intensity (*r* = 0.34, *P* < 0.001), and expected pain intensity (*r* = 0.27, *P* = 0.001), suggesting consistent associations between body image distortion and pain-related perceptions. Furthermore, although weaker, significant correlations were found between FreBAQ_PL scores and emotional distress variables, including pain catastrophizing (*r* = 0.30, *P* < 0.001) and general fear (*r* = 0.23, *P* = 0.003). All reported correlations remained statistically significant after Bonferroni correction.

## 4. Discussion

This study investigated how experimentally induced muscle pain affects body image and sensory perception. In the injection group, acute pain led to greater lower-back perceptual distortion, larger body drawings, and reduced body satisfaction compared with controls, indicating perceptual and attitudinal changes.

Although most body image-related measures (FreBAQ_PL, perceptual distortion of the lower back, body drawings, BES) captured significant effects of pain, sensory testing measures (pain and tactile thresholds, tactile estimation) remained unaffected. This pattern suggests that acute pain selectively alters body image without affecting basic sensory processing. These findings may reflect higher-order mechanisms, where perceptual, cognitive, and attitudinal dimensions are influenced by pain, even when primary sensory thresholds remain stable. Even short-term nociceptive input can produce significant distortions in the affected region, consistent with meta-analytic evidence that pain intensity, rather than duration, is linked to distorted body image in clinical populations.^6^

Assessment of 3 groups—control, sham, and injection—helped separate the effects of simply expecting or feeling an injection from actual short-term pain. Obtained findings suggest that it is not the anticipation or sensation of an injection that alters body image, but rather the intensity of pain itself. Because no body image distortion was observed in the sham group, it seems that mild or brief sensations are not enough to change how the body is perceived—more intense pain appears necessary to affect body image.

The obtained results emphasize that even brief, experimentally induced pain can immediately alter body image, particularly shape and size perception. In the current study, the injection group showed higher scores on the FreBAQ_PL, perceptual distortion, and drawing tasks, indicating perception of the affected area as larger. Similar alterations occur in conditions affecting body size and shape perception (both in chronic and acute pain). In a study of patients with osteoarthritis (OA), individuals reported feeling their body part as swollen and larger, despite the absence of objective indicators.^42^ Similarly, in the context of orofacial pain, individuals with pain at a specific body site, perceived it as larger compared with individuals without pain.^12,13^ This finding supports the concept of somatosensory magnification, in which pain can expand the perceived size of the body, a phenomenon linked to neuroplastic changes in the somatosensory cortex.^26^ Neuroimaging studies have provided further evidence for this effect, demonstrating that pain can modulate the activity in the primary somatosensory cortex, resulting in changes in body part representation.^17^

In line with these observations, studies using visual methods, such as body outline drawings, have demonstrated that individuals with chronic pain perceive alterations in their body shape.^31,33^ The present study used body drawings and found that participants only perceived and pictured their back as larger during acute pain when the assessment was conducted on the right side. This interesting result suggests that the right side, being the dominant side in the studied population, may be more accurately represented in the somatosensory system. This aligns with research demonstrating that the dominant hand exhibits enhanced tactile sensitivity and discrimination^1^ as well as higher pain tolerance compared with the nondominant hand.^36^ The combination of such findings may suggest that frequent use and greater somatosensory input contribute to a more refined sensory representation on the dominant side and, therefore, enables a more refined detection of changes in perceived body size and shape.

Previous studies using the FreBAQ_PL, focused on distorted body image in individuals with chronic pain.^5,7,14,29,30,46,51^ Chronic pain is considered to potentially induce changes in body image, possibly because of cortical reorganization.^16,26,48^ In contrast, the present study induced acute pain to isolate the effects of nociception from other factors. The body image distortions observed during acute pain seemed to be immediate but temporary, resolving as the nociceptive stimulus subsided. This finding aligns with Graven-Nielsen,^19^ suggesting that acute pain-induced changes subsided once the nociceptive stimulus was removed.

This suggests that, unlike chronic pain, acute pain contributes to perceptual changes without permanent changes in the sensory representation of the brain. As acute pain does not induce cortical reorganization, the observed body image changes are likely driven by higher-order mechanisms—such as attention, affective evaluation, and top-down processing—rather than lasting changes in somatosensory maps. Thus, unlike chronic pain, acute pain may alter body image through cognitive-affective pathways without structural neural changes, as also reflected in intact sensory testing.

Experimentally induced acute pain did not affect detection or pain thresholds. The lack of changes in electrical thresholds and TPE suggests that experimental pain induces changes in body image without altering the processing of sensory input from the periphery to higher neural caters in the brain. Previous studies revealed mixed findings. For instance, although altered 2-point discrimination was observed, TPE remained unaffected, as was the case in the present study.^3^ This suggests that pain experience does not impair the ability to position 2 locations of external stimuli and estimate their spatial relationship. However, this negative finding should be interpreted with caution.

The decrease in BES scores in the injection group suggests that acute pain can rapidly and causally reduce body satisfaction, with satisfaction improving as pain subsided. This pattern was absent in the control conditions and may indicate that body satisfaction is closely and causally related to painful experience. This findings are consistent with previous correlational studies linking body satisfaction and pain perception.^27,41,44^

In addition, at baseline, the injection group exhibited the highest weight satisfaction, as captured by the weight discrepancy index (near-zero discrepancy), whereas controls wished to weigh 1.8 to 3.95 kg less. Despite this, a small but significant change in body satisfaction over time occurred only in the injection group, suggesting effects of both time and condition. Weight discrepancy also interacted with FreBAQ_PL and perceptual distortion of the lower back, indicating that weight dissatisfaction may modulate body image responses during pain. This relationship may be partly mediated by broader emotional factors—negative affect, fear, or catastrophizing—consistent with correlations between body image distortion and emotional distress.

Emotional distress variables—general fear, pain catastrophizing, and pain expectations—were all significantly associated with body image distortion (FreBAQ_PL). This aligns with findings by Wand et al.,^47^ who showed that fear and pain expectations contributed to altered body perception in individuals with chronic pain.

The present study found a positive correlation between general fear and FreBAQ_PL, suggesting that individuals with higher anxiety were more prone to body image distortion during acute pain. This aligns with research showing that fear and anxiety can heighten pain attention and disrupt body perception.^11,45^

Pain catastrophizing also showed a significant positive correlation with FreBAQ_PL, highlighting the psychological impact on body perception. Catastrophizing, involving exaggerated negative thoughts and feelings about pain, has been linked to altered body image and increased pain severity and its impact on body perception.^28,38^

Expected injection pain positively correlated with FreBAQ_PL, highlighting the role of anticipatory cognitive processes in modulating body image distortion. Expectations about pain intensity have been shown to influence not only the subjective experience of pain but also associated perceptual and emotional responses.^4,49^

Notably, the injection group showed considerable interindividual variability—reflected in larger standard deviations—which may indicate that individual differences in pain responses modulate the extent of body image distortion, further reinforcing the ecological validity of the findings. Taken together, these results suggest that fear, catastrophizing, and pain-related expectations contribute to body image distortion during acute pain, although general fear did not significantly interact with other variables, implying it may not be a key contributor to body image distortion.

## 5. Limitations

The study provides novel causal evidence linking pain to changes in body image. However, it focuses on the immediate effects of acute pain, leaving the long-term trajectory of these changes unclear. Future research could explore whether distortions persist or fluctuate over time and how chronic pain may differently affect body perception and emotional distress.

Although our study provides a controlled model for examining pain-related changes in body image, using healthy participants under experimentally induced pain limits generalization to clinical populations. However, this model offers a valuable opportunity to isolate the effects of nociceptive input from long-term cognitive, emotional, behavioral, or biological factors that often co-occur in chronic pain. Identifying acute, pain-driven distortions without pathology may help generate mechanistic hypotheses and guide early interventions to prevent body image distortion in chronic pain.

## 6. Conclusions

Key findings demonstrate that acute pain significantly increased body image distortion, reduced body satisfaction, and altered perceptions of size and shape. The evidence for perception of the painful body region as larger, further supports the concept of somatosensory magnification.

Sensory testing remained stable across groups, indicating intact biological sensory functions, whereas acute pain was strongly associated with psychological and perceptual variables, including general fear, pain catastrophizing, and pain expectations. These results highlight the role of psychological processes in modulating pain and its impact on body perception and emphasize that body image distortion in acute pain is driven more by perceptual and emotional factors, while highlighting the limited role of sensory testing in these processes.

## Conflict of interest statement

The authors have no conflicts of interest to declare.

## Appendix A. Supplemental digital content

Supplemental digital content associated with this article can be found online at http://links.lww.com/PAIN/C420.

## Supplementary Material

SUPPLEMENTARY MATERIAL
