# Supplementary material for: Perceiving pain alters body perception: the effects of acute pain on body image and sensory testing
Source: Pain. 2025 Nov 26;167(4):776–85. doi: 10.1097/j.pain.0000000000003872 (PMC12994510; doi:10.1097/j.pain.0000000000003872)
Supplement: SUPPLEMENTARY MATERIAL [file jop-167-776-s002.pdf]

Supplementary File

Table of contents:

Supplementary Table 1. Demographic Characteristics and Baseline Differences Between Groups
Supplementary Table 2. Effects of Experimental Manipulation on FreBAQ
Supplementary Table 2a. Effects of Experimental Manipulation on FreBAQ with Covariates [GF and WDI]
Supplementary Table 2b. Effects of Experimental Manipulation on FreBAQ - Pairwise Comparisons (between groups)
Supplementary Table 2c. Effects of Experimental Manipulation on FreBAQ - Pairwise Comparisons (between assessments)
Supplementary Table 3. Effects of Experimental Manipulation on PT (Pain Threshold)
Supplementary Table 3a. Effects of Experimental Manipulation on PT with Covariates [GF and WDI]
Supplementary Table 3b. Effects of Experimental Manipulation on PT - Pairwise Comparisons (between groups)
Supplementary Table 3c. Effects of Experimental Manipulation on PT - Pairwise Comparisons (between assessments)
Supplementary Table 4. Effects of Experimental Manipulation on DT (Detection Threshold)
Supplementary Table 4a. Effects of Experimental Manipulation on DT with Covariates [GF and WDI]
Supplementary Table 4b. Effects of Experimental Manipulation on DT - Pairwise Comparisons (between groups)
Supplementary Table 4c. Effects of Experimental Manipulation on DT - Pairwise Comparisons (between assessments)
Supplementary Table 5. Effects of Experimental Manipulation on TPE_V (Two Point Estimation – Vertical)
Supplementary Table 5a. Effects of Experimental Manipulation on TPE_V with Covariates [GF and WDI]
Supplementary Table 5b. Effects of Experimental Manipulation on TPE_V - Pairwise Comparisons (between groups)
Supplementary Table 5c. Effects of Experimental Manipulation on TPE_V - Pairwise Comparisons (between assessments)
Supplementary Table 6. Effects of Experimental Manipulation on TPE_H (Two Point Estimation – Horizontal)
Supplementary Table 6a. Effects of Experimental Manipulation on TPE_H with Covariates [GF and WDI]
Supplementary Table 6b. Effects of Experimental Manipulation on TPE_H - Pairwise Comparisons (between groups)
Supplementary Table 6c. Effects of Experimental Manipulation on TPE_H - Pairwise Comparisons (between assessments)
Supplementary Table 7. Effects of Experimental Manipulation on FE (Finger Estimation)
Supplementary Table 7a. Effects of Experimental Manipulation on FE with Covariates [GF and WDI]
Supplementary Table 7b. Effects of Experimental Manipulation on FE - Pairwise Comparisons (between groups)
Supplementary Table 7c. Effects of Experimental Manipulation on FE - Pairwise Comparisons (between assessments)
Supplementary Table 8. Effects of Experimental Manipulation on APD (Area of Perceived Distortion)
Supplementary Table 8a. Effects of Experimental Manipulation on APD with Covariates [GF and WDI]
Supplementary Table 8b. Effects of Experimental Manipulation on APD - Pairwise Comparisons (between groups)
Supplementary Table 8c. Effects of Experimental Manipulation on APD - Pairwise Comparisons (between assessments)
Supplementary Table 9. Effects of Experimental Manipulation on MES (Magnitude Estimation Scale)
Supplementary Table 9a. Effects of Experimental Manipulation on MES with Covariates [GF and WDI]
Supplementary Table 9b. Effects of Experimental Manipulation on MES - Pairwise Comparisons (between groups)
Supplementary Table 9c. Effects of Experimental Manipulation on MES - Pairwise Comparisons (between assessments)

Supplementary Table 10. Effects of Experimental Manipulation on DR_R (Back Drawing - Right Side)
Supplementary Table 10a. Effects of Experimental Manipulation on DR_R with Covariates [GF and WDI]
Supplementary Table 10b. Effects of Experimental Manipulation on DR_R - Pairwise Comparisons (between groups)
Supplementary Table 10c. Effects of Experimental Manipulation on DR_R - Pairwise Comparisons (between assessments)
Supplementary Table 11. Effects of Experimental Manipulation on DR_L (Back Drawing - Left Side)
Supplementary Table 11a. Effects of Experimental Manipulation on DR_L with Covariates [GF and WDI]
Supplementary Table 11b. Effects of Experimental Manipulation on DR_L - Pairwise Comparisons (between groups)
Supplementary Table 11c. Effects of Experimental Manipulation on DR_L - Pairwise Comparisons (between assessments)

Supplementary Table 12. Effects of Experimental Manipulation on BES (Body Esteem Scale)
Supplementary Table 12a. Effects of Experimental Manipulation on BES with Covariates [GF and WDI]
Supplementary Table 12b. Effects of Experimental Manipulation on BES - Pairwise Comparisons (between groups)
Supplementary Table 12c. Effects of Experimental Manipulation on BES - Pairwise Comparisons (between assessments)

Supplementary Table 13. Summary of All Variables Across Groups and Time Points

Supplementary Table 1. Demographic description of the population and the baseline differences

	Injection N=31		*Sham* N=30		*Control* N=30		*Test statistic*		
**Variable**	M	SD	M	SD	M	SD	F	p	η^2
**Age, years**	22,26	1,73	22,80	3,83	22,97	4,33	0,35	0,71	0,01
**Height, meters**	184,35	51,58	172,78	8,48	176,43	10,04	1,11	0,33	0,02
**Weight actual, kg**	70,44	11,96	73,39	14,73	72,68	11,45	0,44	0,64	0,01
**Weight desired, kg**	70,94	13,06	69,43	12,93	70,88	12,26	0,13	0,87	0,00
**BMI**	22,42	4,92	24,46	3,81	23,23	2,10	0,13	0,11	0,05
**WDI**	0,49	1,27	-3,95	1,29	-1,8	1,29	3,02	0,05*	0,06
**GF**	38,26	21,35	34,67	18,11	25,40	18,93	3,50	0,03**	0,07
**IF**	40,26	29,59	33,03	23,33	29,97	19,69	1,41	0,25	0,03
**Expectancy**	44,39	24,24	35,13	20,32	33,57	22,08	2,21	0,12	0,05
**PCS**	12,61	9,09	14,33	9,79	9,57	7,07	2,29	0,11	0,05
			Number of participants						
**Sex (Women/Man)**	13/18		16/14		12/18				
**Laterality (R)**	30		29		29				
**Whether injection (Y)**	30		22		0				

LEGEND: N – number of participants, M – mean, SD – standard deviation, F – statistic, η^2 – partial eta squared, BMI – body mass index, WDI – weight discrepancy index, GF – general fear, IF – injection fear, Expectancy – pain injection expectancy, PCS – pain catastrophizing scale, R – right-handed individuals

Supplementary Table 2. The effects of experimental manipulation on FreBAQ: main effect (interaction between assessment and group), effect of assessment and effect of group.

	SS	*df*	**Mean Square**	*F*	*p*	η^2
			**Total sample (*N*=91)**			
Assessment	150,42	1,65	91,27	9,21	<,001*	,095
Assessment * Group	326,23	3,30	98,98	9,99	<,001*	,185
Error (Phase)	1436,59	145,03	9,91			
Group	229,77	2	114,89	2,33	,10	,050

LEGEND: SS - Type III Sum of Squares, df - degrees of freedom, η^2 - partial eta squared

Supplementary Table 2a. The effects of experimental manipulation on FreBAQ: main effect (interaction between assessment and group), effect of assessment and effect of group with covariants (WDI and GF)

	SS	*df*	**Mean Square**	*F*	*p*	η^2
			**Total sample (*N*=91)**			
Assessment	3,34	1,66	2,01	0,21	,768	,002
Assessment * GF	19,51	1,66	11,71	1,24	,287	,014
Assessment * WDI	61,33	1,66	36,80	3,90	,029*	,043
Assessment * Group	329,44	3,33	98,84	10,49	<,001*	,196
Error (Phase)	1350,96	143,32	9,43			
Group	189,52	2	94,76	1,91	,16	,042

LEGEND: SS - Type III Sum of Squares, df - degrees of freedom, η^2 - partial eta squared, WDI – weight discrepancy index, GF – general fear

Supplementary Table 2b. The effects of experimental manipulation on FreBAQ: results of pairwise comparisons (between groups in each assessment)

Assessment	Group	Group	MD	SD	p	95% CI	
						Lower	Upper
1	Injection	Control	-0,89	1,11	1,00	-3,59	1,82
		Sham	-0,64	1,10	1,00	-3,31	2,04
	Control	Injection	0,89	1,11	1,00	-1,82	3,59
		Sham	0,25	1,09	1,00	-2,41	2,91
	Sham	Injection	0,64	1,10	1,00	-2,04	3,31
		Control	-0,25	1,09	1,00	-2,91	2,41
2	Injection	Control	4,75	1,43	0,00*	1,26	8,24
		Sham	5,39	1,42	<,001*	1,94	8,85
	Control	Injection	-4,75	1,43	0,00*	-8,24	-1,26
		Sham	0,65	1,41	1,00	-2,79	4,08
	Sham	Injection	-5,39	1,42	<,001*	-8,85	-1,94
		Control	-0,65	1,41	1,00	-4,08	2,79
3	Injection	Control	1,02	1,21	1,00	-1,93	3,96
		Sham	1,25	1,19	0,90	-1,67	4,16
	Control	Injection	-1,02	1,21	1,00	-3,96	1,93
		Sham	0,23	1,19	1,00	-2,66	3,13
	Sham	Injection	-1,25	1,19	0,90	-4,16	1,67
		Control	-0,23	1,19	1,00	-3,13	2,66

LEGEND: MD - Mean difference SD - Standard Deviation, CI - confidence interval

Supplementary Table 2c. The effects of experimental manipulation on FreBAQ: results of pairwise comparisons (between assessments in each group)

Assessment	Group	Group	MD	SD	p	95% CI	
						Lower	Upper
Injection	1	2	-5,18	0,88	<,001*	-7,34	-3,03
		3	-0,82	0,68	0,72	-2,47	0,86
	2	1	5,18	0,88	<,001*	3,03	7,34
		3	4,38	0,62	<,001*	2,87	5,88
	3	1	0,81	0,68	0,72	-0,86	2,47
		2	-4,38	0,62	<,001*	-5,88	-2,87
Control	1	2	0,45	0,89	1,00	-1,73	2,62
		3	1,09	0,69	0,35	-0,59	2,77
	2	1	-0,45	0,89	1,00	-2,62	1,73
		3	0,65	0,62	0,91	-0,87	2,16
	3	1	-1,09	0,69	0,35	-2,77	0,59
		2	-0,65	0,62	0,91	-2,16	0,87
Sham	1	2	0,84	0,88	1,00	-1,31	3,00
		3	1,08	0,68	0,36	-0,59	2,74
	2	1	-0,84	0,88	1,00	-3,00	1,31
		3	0,23	0,62	1,00	-1,28	1,74
	3	1	-1,08	0,68	0,36	-2,74	0,59
		2	-0,23	0,62	1,00	-1,74	1,28

LEGEND: MD - Mean difference SD - Standard Deviation, CI - confidence interval

Supplementary Table 3. The effects of experimental manipulation on PT: interaction between assessment and group), effect of assessment and effect of group

	SS	*df*	**Mean Square**	*F*	*p*	*η²*
			**Total sample (*N*=91)**			
**Assessment**	106,86	1,31	81,64	29,76	<,001*	0,253
**Assessment * Group**	1,49	2,62	0,57	0,21	0,867	0,005
Error (Phase)	315,97	115,19	2,74			
Group	1,59	2	0,80	0,47	,954	0,001

LEGEND: SS - Type III Sum of Squares, df - degrees of freedom, η^2 - partial eta squared

Supplementary Table 3a. The effects of experimental manipulation on PT: main effect (interaction between assessment and group), effect of assessment and effect of group with covariants (WDI and GF)

	SS	*df*	**Mean Square**	*F*	*p*	*η²*
			**Total sample (*N*=91)**			
Assessment	45,46	1,30	34,91	12,77	<,001*	0,129
Assessment * GF	4,20	1,30	3,22	1,18	0,294	0,005
Assessment * WDI	6,38	1,30	4,90	1,79	0,182	0,02
Assessment * Group	2,38	2,60	0,91	0,33	0,772	0,008
Error (Phase)	306,08	111,99	2,73			
Group	5,37	2	2,685	0,16	,852	0,004

LEGEND: SS - Type III Sum of Squares, df - degrees of freedom, η^2 - partial eta squared, WDI – weight discrepancy index, GF – general fear

Supplementary Table 3b. The effects of experimental manipulation on PT: results of pairwise comparisons (between groups in each assessment)

**Assessment**	**Group**	**Group**	**MD**	**SD**	**p**	**95% CI**	
						**Lower**	**Upper**
1	Injection	Control	0,36	0,84	1,00	-1,70	2,41
		Sham	-0,06	0,83	1,00	-2,10	1,98
	Control	Injection	-0,36	0,84	1,00	-2,41	1,70
		Sham	-0,42	0,83	1,00	-2,44	1,61
	Sham	Injection	0,06	0,83	1,00	-1,98	2,10
		Control	0,42	0,83	1,00	-1,61	2,44
2	Injection	Control	-0,15	0,64	1,00	-1,71	1,41
		Sham	-0,50	0,63	1,00	-2,04	1,05
	Control	Injection	0,15	0,64	1,00	-1,41	1,71
		Sham	-0,35	0,63	1,00	-1,88	1,19
	Sham	Injection	0,50	0,63	1,00	-1,05	2,04
		Control	0,35	0,63	1,00	-1,19	1,88
3	Injection	Control	-0,04	0,59	1,00	-1,48	1,40
		Sham	-0,27	0,58	1,00	-1,69	1,16
	Control	Injection	0,04	0,59	1,00	-1,40	1,48
		Sham	-0,23	0,58	1,00	-1,64	1,19
	Sham	Injection	0,27	0,58	1,00	-1,16	1,69
		Control	0,23	0,58	1,00	-1,19	1,64

LEGEND: MD - Mean difference SD - Standard Deviation, CI - confidence interval

Supplementary Table 3c. The effects of experimental manipulation on PT: results of pairwise comparisons (between assessments in each group)

Assessment	Group	Group	MD	SD	p	95% CI	
						Lower	Upper
Injection	1	2	1,60	0,39	<,001*	0,65	2,56
		3	1,56	0,43	0,00*	0,53	2,60
	2	1	-1,60	0,39	<,001*	-2,56	-0,65
		3	-0,04	0,19	1,00	-0,49	0,41
	3	1	-1,56	0,43	0,00*	-2,60	-0,53
		2	0,04	0,19	1,00	-0,41	0,49
Control	1	2	1,09	0,40	0,02*	0,13	2,06
		3	1,16	0,43	0,02*	0,12	2,21
	2	1	-1,09	0,40	0,02*	-2,06	-0,13
		3	0,07	0,19	1,00	-0,39	0,52
	3	1	-1,16	0,43	0,02*	-2,21	-0,12
		2	-0,07	0,19	1,00	-0,52	0,39
Sham	1	2	1,17	0,39	0,01*	0,21	2,12
		3	1,36	0,43	0,01*	0,32	2,39
	2	1	-1,17	0,39	0,01*	-2,12	-0,21
		3	0,19	0,19	0,93	-0,26	0,64
	3	1	-1,36	0,43	0,01*	-2,39	-0,32
		2	-0,19	0,19	0,93	-0,64	0,26

LEGEND: MD - Mean difference SD - Standard Deviation, CI - confidence interval

Supplementary Table 4. The effects of experimental manipulation on DT: main effect (interaction between assessment and group), effect of assessment and effect of group.

	SS	*df*	Mean Square	*F*	*p*	*η²*
**Total sample (*N*=91)**						
Assessment	0,20	1,74	0,12	3,35	0,044*	0,037
Assessment * Group	0,08	3,47	0,02	0,70	0,573	0,016
Error (Phase)	5,22	152,70	0,03			
Group	0,27	2	0,14	0,47	,629	0,010

LEGEND: SS - Type III Sum of Squares, df - degrees of freedom, η^2 - partial eta squared

Supplementary Table 4a. The effects of experimental manipulation on DT: main effect (interaction between assessment and group), effect of assessment and effect of group with covariants (WDI and GF)

	SS	*df*	Mean Square	*F*	*p*	*η²*
**Total sample (*N*=91)**						
Assessment	0,09	1,72	0,05	1,44	0,24	0,016
Assessment * GF	0,03	1,72	0,02	0,44	0,612	0,005
Assessment * WDI	0,12	1,72	0,07	1,97	0,149	0,022
Assessment * Group	0,07	3,43	0,02	0,55	0,673	0,013
Error (Phase)	5,08	147,54	0,03			
Group	0,29	2	0,144	0,49	,615	0,011

LEGEND: SS - Type III Sum of Squares, df - degrees of freedom, η^2 - partial eta squared, WDI – weight discrepancy index, GF – general fear

Supplementary Table 4b. The effects of experimental manipulation on DT: results of pairwise comparisons (between groups in each assessment)

Assessment	Group	Group	MD	SD	p	95% CI	
						Lower	Upper
1	Injection	Control	0,05	0,10	1,00	-0,18	0,29
		Sham	0,05	0,10	1,00	-0,19	0,28
	Control	Injection	-0,05	0,10	1,00	-0,29	0,18
		Sham	-0,01	0,10	1,00	-0,24	0,23
	Sham	Injection	-0,05	0,10	1,00	-0,28	0,19
		Control	0,01	0,10	1,00	-0,23	0,24
2	Injection	Control	0,10	0,09	0,86	-0,13	0,32
		Sham	0,13	0,09	0,50	-0,10	0,35
	Control	Injection	-0,10	0,09	0,86	-0,32	0,13
		Sham	0,03	0,09	1,00	-0,19	0,25
	Sham	Injection	-0,13	0,09	0,50	-0,35	0,10
		Control	-0,03	0,09	1,00	-0,25	0,19
3	Injection	Control	0,03	0,09	1,00	-0,18	0,24
		Sham	0,06	0,09	1,00	-0,15	0,28
	Control	Injection	-0,03	0,09	1,00	-0,24	0,18
		Sham	0,03	0,09	1,00	-0,18	0,25
	Sham	Injection	-0,06	0,09	1,00	-0,28	0,15
		Control	-0,03	0,09	1,00	-0,25	0,18

LEGEND: MD - Mean difference SD - Standard Deviation, CI - confidence interval

Supplementary Table 4c. The effects of experimental manipulation on DT: results of pairwise comparisons (between assessments in each group)

Assessment	Group	Group	MD	SD	p	95% CI	
						**Lower**	**Upper**
Injection	1	2	-0,01	0,05	1,00	-0,14	0,12
		3	0,07	0,04	0,25	-0,03	0,16
	2	1	0,01	0,05	1,00	-0,12	0,14
		3	0,08	0,04	0,21	-0,03	0,18
	3	1	-0,07	0,04	0,25	-0,16	0,03
		2	-0,08	0,04	0,21	-0,18	0,03
Control	1	2	0,04	0,05	1,00	-0,10	0,17
		3	0,04	0,04	0,81	-0,05	0,14
	2	1	-0,04	0,05	1,00	-0,17	0,10
		3	0,01	0,04	1,00	-0,10	0,11
	3	1	-0,04	0,04	0,81	-0,14	0,05
		2	-0,01	0,04	1,00	-0,11	0,10
Sham	1	2	0,07	0,05	0,55	-0,06	0,20
		3	0,09	0,04	0,10	-0,01	0,18
	2	1	-0,07	0,05	0,55	-0,20	0,06
		3	0,01	0,04	1,00	-0,09	0,12
	3	1	-0,09	0,04	0,10	-0,18	0,01
		2	-0,01	0,04	1,00	-0,12	0,09

LEGEND: MD - Mean difference SD - Standard Deviation, CI - confidence interval

Supplementary Table 5. The effects of experimental manipulation on TPE_V: main effect (interaction between assessment and group), effect of assessment and effect of group.

	SS	*df*	Mean Square	*F*	*p*	*η²*
**Total sample (*N*=91)**						
Assessment	5693,57	1,68	3395,34	11,92	<,001*	0,119
Assessment * Group	1407,31	3,35	419,62	1,47	0,221	0,032
Error (Phase)	42046,31	147,57	284,93			
Group	4552,07	2,00	2276,03	1,73	0,18	0,038

LEGEND: SS - Type III Sum of Squares, df - degrees of freedom, η^2 - partial eta squared

Supplementary Table 5a. The effects of experimental manipulation on TPE_V: main effect (interaction between assessment and group), effect of assessment and effect of group with covariants (WDI and GF)

	SS	*df*	Mean Square	*F*	*p*	*η²*
**Total sample (*N*=91)**						
Assessment	892,37	1,68	532,07	1,83	0,17	0,021
Assessment * GF	108,85	1,68	64,90	0,22	0,761	0,003
Assessment * WDI	91,27	1,68	54,42	0,19	0,791	0,002
Assessment * Group	1513,82	3,35	451,31	1,56	0,198	0,035
Error (Phase)	41857,11	144,24	290,20			
Group	5403,98	2	2701,99	2,04	,136	0,045

LEGEND: SS - Type III Sum of Squares, df - degrees of freedom, η^2 - partial eta squared, WDI – weight discrepancy index, GF – general fear

Supplementary Table 5b. The effects of experimental manipulation on TPE_V: results of pairwise comparisons (between groups in each assessment)

Assessment	Group	Group	MD	SD	p	95% CI	
						Lower	Upper
1	Injection	Control	-3,68	6,79	1,00	-20,26	12,90
		Sham	-0,67	6,72	1,00	-17,08	15,74
	Control	Injection	3,68	6,79	1,00	-12,90	20,26
		Sham	3,01	6,68	1,00	-13,31	19,32
	Sham	Injection	0,67	6,72	1,00	-15,74	17,08
		Control	-3,01	6,68	1,00	-19,32	13,31
2	Injection	Control	-17,21	6,48	0,03*	-33,02	-1,40
		Sham	-8,63	6,41	0,54	-24,28	7,02
	Control	Injection	17,21	6,48	0,03*	1,40	33,02
		Sham	8,57	6,37	0,55	-6,98	24,13
	Sham	Injection	8,63	6,41	0,54	-7,02	24,28
		Control	-8,57	6,37	0,55	-24,13	6,98
3	Injection	Control	-13,29	6,53	0,14	-29,24	2,66
		Sham	-9,65	6,47	0,42	-25,44	6,13
	Control	Injection	13,29	6,53	0,14	-2,66	29,24
		Sham	3,64	6,43	1,00	-12,06	19,33
	Sham	Injection	9,65	6,47	0,42	-6,13	25,44
		Control	-3,64	6,43	1,00	-19,33	12,06

LEGEND: MD - Mean difference SD - Standard Deviation, CI - confidence interval

Supplementary Table 5c. The effects of experimental manipulation on TPE_V: results of pairwise comparisons (between assessments in each group)

Assessment	Group	Group	MD	SD	p	95% CI	
						**Lower**	**Upper**
Injection	1	2	-1,54	4,47	1,00	-12,44	9,37
		3	-4,26	4,58	1,00	-15,44	6,93
	2	1	1,54	4,47	1,00	-9,37	12,44
		3	-2,72	3,07	1,00	-10,22	4,79
	3	1	4,26	4,58	1,00	-6,93	15,44
		2	2,72	3,07	1,00	-4,79	10,22
Control	1	2	-15,06	4,50	0,00*	-26,05	-4,08
		3	-13,87	4,61	0,01*	-25,13	-2,60
	2	1	15,06	4,50	0,00*	4,08	26,05
		3	1,20	3,10	1,00	-6,36	8,76
	3	1	13,87	4,61	0,01*	2,60	25,13
		2	-1,20	3,10	1,00	-8,76	6,36
Sham	1	2	-9,50	4,47	0,11	-20,40	1,41
		3	-13,23	4,58	0,02*	-24,42	-2,05
	2	1	9,50	4,47	0,11	-1,41	20,40
		3	-3,74	3,07	0,68	-11,24	3,77
	3	1	13,23	4,58	0,02*	2,05	24,42
		2	3,74	3,07	0,68	-3,77	11,24

LEGEND: MD - Mean difference SD - Standard Deviation, CI - confidence interval

Supplementary Table 6. The effects of experimental manipulation on TPE_H: main effect (interaction between assessment and group), effect of assessment and effect of group.

	SS	*df*	Mean Square	*F*	*p*	η^2
**Total sample (*N*=91)**						
Assessment	4498,43	1,93	2331,98	12,08	<,001*	0,121
Assessment * Group	56,95	3,86	14,76	0,08	0,988	0,005
Error (Phase)	32764,71	169,75	193,01			
Group	1722,41	2,00	861,21	0,53	0,591	0,012

LEGEND: SS - Type III Sum of Squares, df - degrees of freedom, η^2 - partial eta squared

Supplementary Table 6a. The effects of experimental manipulation on TPE_H: main effect (interaction between assessment and group), effect of assessment and effect of group with covariants (WDI and GF)

	SS	*df*	Mean Square	*F*	*p*	η^2
**Total sample (*N*=91)**						
Assessment	1058,31	1,93	548,90	2,82	0,064	0,032
Assessment * GF	266,05	1,93	137,99	0,71	0,489	0,008
Assessment * WDI	228,89	1,93	118,71	0,61	0,539	0,007
Assessment * Group	126,39	3,86	32,78	0,17	0,95	0,004
Error (Phase)	32274,76	165,81	194,65			
Group	512,96	2,00	256,48	0,19	0,83	0,009

LEGEND: SS - Type III Sum of Squares, df - degrees of freedom, η^2 - partial eta squared, WDI – weight discrepancy index, GF – general fear

Supplementary Table 6b. The effects of experimental manipulation on TPE_H: results of pairwise comparisons (between groups in each assessment)

Assessment	Group	Group	MD	SD	p	95% CI	
						Lower	Upper
1	Injection	Control	-5,79	6,32	1,00	-21,22	9,64
		Sham	-10,11	6,25	0,33	-25,38	5,16
	Control	Injection	5,79	6,32	1,00	-9,64	21,22
		Sham	-4,32	6,22	1,00	-19,50	10,86
	Sham	Injection	10,11	6,25	0,33	-5,16	25,38
		Control	4,32	6,22	1,00	-10,86	19,50
2	Injection	Control	-5,82	7,14	1,00	-23,25	11,61
		Sham	-9,17	7,07	0,59	-26,42	8,09
	Control	Injection	5,82	7,14	1,00	-11,61	23,25
		Sham	-3,34	7,02	1,00	-20,49	13,81
	Sham	Injection	9,17	7,07	0,59	-8,09	26,42
		Control	3,34	7,02	1,00	-13,81	20,49
3	Injection	Control	-2,40	7,12	1,00	-19,79	14,98
		Sham	-8,84	7,05	0,64	-26,05	8,37
	Control	Injection	2,40	7,12	1,00	-14,98	19,79
		Sham	-6,44	7,01	1,00	-23,54	10,67
	Sham	Injection	8,84	7,05	0,64	-8,37	26,05
		Control	6,44	7,01	1,00	-10,67	23,54

LEGEND: MD - Mean difference SD - Standard Deviation, CI - confidence interval

Supplementary Table 6c. The effects of experimental manipulation on TPE_H: results of pairwise comparisons (between assessments in each group)

Assessment	Group	Group	MD	SD	p	95% CI	
						Lower	Upper
Injection	1	2	-9,23	3,23	0,02*	-17,13	-1,34
		3	-9,79	3,78	0,03*	-19,03	-0,55
	2	1	9,23	3,23	0,02*	1,34	17,13
		3	-0,56	3,75	1,00	-9,71	8,60
	3	1	9,79	3,78	0,03*	0,55	19,03
		2	0,56	3,75	1,00	-8,60	9,71
Control	1	2	-9,27	3,26	0,02*	-17,22	-1,32
		3	-6,41	3,81	0,29	-15,72	2,90
	2	1	9,27	3,26	0,02*	1,32	17,22
		3	2,86	3,78	1,00	-6,37	12,08
	3	1	6,41	3,81	0,29	-2,90	15,72
		2	-2,86	3,78	1,00	-12,08	6,37
Sham	1	2	-8,29	3,23	0,04*	-16,19	-0,40
		3	-8,52	3,79	0,08	-17,77	0,72
	2	1	8,29	3,23	0,04*	0,40	16,19
		3	-0,23	3,75	1,00	-9,39	8,93
	3	1	8,52	3,79	0,08	-0,72	17,77
		2	0,23	3,75	1,00	-8,93	9,39

LEGEND: MD - Mean difference SD - Standard Deviation, CI - confidence interval

Supplementary Table 7. The effects of experimental manipulation on Finger Estimation [FE]: main effect (interaction between assessment and group), effect of assessment and effect of group.

	SS	*df*	Mean Square	*F*	*p*	η^2
**Total sample (*N*=91)**						
Assessment	3,71	1,46	2,54	9,50	<,001*	0,097
Assessment * Group	0,53	2,92	0,18	0,68	0,562	0,015
Error (Phase)	34,39	176,00	0,20			
Group	15,53	2	7,76	1,04	,356	0,023

LEGEND: SS - Type III Sum of Squares, df - degrees of freedom, η^2 - partial eta squared

Supplementary Table 7a. The effects of experimental manipulation on Finger Estimation [FE]: main effect (interaction between assessment and group), effect of assessment and effect of group with covariants (WDI and GF)

	SS	*df*	Mean Square	*F*	*p*	η^2
**Total sample (*N*=91)**						
Assessment	2,11	1,46	1,45	5,33	0,012*	0,058
Assessment * GF	0,39	1,46	0,27	0,98	0,356	0,011
Assessment * WDI	0,00	1,46	0,00	0,00	0,994	0
Assessment * Group	0,75	2,91	0,26	0,95	0,418	0,022
Error (Phase)	34,00	125,17	0,27			
Group	7,85	2	3,93	0,54	,587	0,012

LEGEND: SS - Type III Sum of Squares, df - degrees of freedom, η^2 - partial eta squared, WDI – weight discrepancy index, GF – general fear

Supplementary Table 7b. The effects of experimental manipulation on Finger Estimation [FE]: results of pairwise comparisons (between groups in each assessment)

Assessment	Group	Group	MD	SD	p	95% CI	
						Lower	Upper
1	Injection	Control	0,16	0,43	1,00	-0,89	1,22
		Sham	-0,17	0,43	1,00	-1,21	0,87
	Control	Injection	-0,16	0,43	1,00	-1,22	0,89
		Sham	-0,33	0,43	1,00	-1,37	0,71
	Sham	Injection	0,17	0,43	1,00	-0,87	1,21
		Control	0,33	0,43	1,00	-0,71	1,37
2	Injection	Control	0,27	0,43	1,00	-0,77	1,31
		Sham	-0,11	0,42	1,00	-1,14	0,92
	Control	Injection	-0,27	0,43	1,00	-1,31	0,77
		Sham	-0,37	0,42	1,00	-1,40	0,65
	Sham	Injection	0,11	0,42	1,00	-0,92	1,14
		Control	0,37	0,42	1,00	-0,65	1,40
3	Injection	Control	0,48	0,44	0,84	-0,59	1,54
		Sham	-0,07	0,43	1,00	-1,12	0,99
	Control	Injection	-0,48	0,44	0,84	-1,54	0,59
		Sham	-0,54	0,43	0,64	-1,59	0,51
	Sham	Injection	0,07	0,43	1,00	-0,99	1,12
		Control	0,54	0,43	0,64	-0,51	1,59

LEGEND: MD - Mean difference SD - Standard Deviation, CI - confidence interval

Supplementary Table 7c. The effects of experimental manipulation on Finger Estimation [FE]: results of pairwise comparisons (between assessments in each group)

Assessment	Group	Group	MD	SD	p	95% CI	
						**Lower**	**Upper**
Injection	1	2	-0,27	0,12	0,10	-0,56	0,03
		3	-,41	0,14	0,01*	-0,76	-0,07
	2	1	0,27	0,12	0,10	-0,03	0,56
		3	-0,15	0,08	0,18	-0,34	0,04
	3	1	,41	0,14	0,01*	0,07	0,76
		2	0,15	0,08	0,18	-0,04	0,34
Control	1	2	-0,16	0,12	0,60	-0,46	0,14
		3	-0,10	0,14	1,00	-0,45	0,25
	2	1	0,16	0,12	0,60	-0,14	0,46
		3	0,06	0,08	1,00	-0,13	0,25
	3	1	0,10	0,14	1,00	-0,25	0,45
		2	-0,06	0,08	1,00	-0,25	0,13
Sham	1	2	-0,20	0,12	0,31	-0,50	0,10
		3	-0,31	0,14	0,10	-0,66	0,04
	2	1	0,20	0,12	0,31	-0,10	0,50
		3	-0,11	0,08	0,50	-0,30	0,08
	3	1	0,31	0,14	0,10	-0,04	0,66
		2	0,11	0,08	0,50	-0,08	0,30

LEGEND: MD - Mean difference SD - Standard Deviation, CI - confidence interval

Supplementary Table 8. The effects of experimental manipulation on APD: main effect (interaction between assessment and group), effect of assessment and effect of group.

	SS	*df*	Mean Square	*F*	*p*	η^2
			**Total sample (*N*=91)**			
Assessment	2,11	1,50	1,41	38,06	<,001*	0,302
Assessment * Group	3,29	3	1,10	29,71	<,001*	0,403
Error (Phase)	4,88	132,06	0,04			
Group	2,39	2	1,19	30,65	<,001*	0,411

LEGEND: SS - Type III Sum of Squares, df - degrees of freedom, η^2 - partial eta squared

Supplementary Table 8a. The effects of experimental manipulation on APD: main effect (interaction between assessment and group), effect of assessment and effect of group with covariants (WDI and GF)

	SS	*df*	Mean Square	*F*	*p*	η^2
			**Total sample (*N*=91)**			
Assessment	0,19	1,52	0,13	3,48	0,046*	0,039
Assessment * GF	0,12	1,52	0,08	2,08	0,141	0,024
Assessment * WDI	0,01	1,52	0,00	0,10	0,848	0,001
Assessment * Group	2,83	3,03	0,94	25,66	<,001*	0,374
Error (Phase)	4,75	130,28	0,04			
Group	2,11	2	1,05	26,88	<,001*	0,385

LEGEND: SS - Type III Sum of Squares, df - degrees of freedom, η^2 - partial eta squared, WDI – weight discrepancy index, GF – general fear

Supplementary Table 8b. The effects of experimental manipulation on APD: results of pairwise comparisons (between groups in each assessment)

Assessment	Group	Group	MD	SD	p	95% CI	
						Lower	Upper
1	Injection	Control	0,00	0,00	.	0,00	0,00
		Sham	0,00	0,00	.	0,00	0,00
	Control	Injection	0,00	0,00	.	0,00	0,00
		Sham	0,00	0,00	.	0,00	0,00
	Sham	Injection	0,00	0,00	.	0,00	0,00
		Control	0,00	0,00	.	0,00	0,00
2	Injection	Control	0,49	0,07	<,001*	0,31	0,66
		Sham	0,53	0,07	<,001*	0,36	0,71
	Control	Injection	-0,49	0,07	<,001*	-0,66	-0,31
		Sham	0,05	0,07	1,00	-0,12	0,22
	Sham	Injection	-0,53	0,07	<,001*	-0,71	-0,36
		Control	-0,05	0,07	1,00	-0,22	0,12
3	Injection	Control	0,07	0,04	0,33	-0,03	0,17
		Sham	0,08	0,04	0,19	-0,02	0,18
	Control	Injection	-0,07	0,04	0,33	-0,17	0,03
		Sham	0,01	0,04	1,00	-0,09	0,11
	Sham	Injection	-0,08	0,04	0,19	-0,18	0,02
		Control	-0,01	0,04	1,00	-0,11	0,09

LEGEND: MD - Mean difference SD - Standard Deviation, CI - confidence interval

Supplementary Table 8c. The effects of experimental manipulation on APD: results of pairwise comparisons (between assessments in each group)

Assessment	Group	Group	MD	SD	p	95% CI	
						Lower	Upper
Injection	1	2	-0,54	0,05	<,001*	-0,66	-0,42
		3	-0,08	0,03	0,02*	-0,15	-0,01
	2	1	0,54	0,05	<,001*	0,42	0,66
		3	0,46	0,05	<,001*	0,34	0,58
	3	1	0,08	0,03	0,02*	0,01	0,15
		2	-0,46	0,05	<,001*	-0,58	-0,34
Control	1	2	-0,05	0,05	0,85	-0,18	0,07
		3	-0,01	0,03	1,00	-0,08	0,06
	2	1	0,05	0,05	0,85	-0,07	0,18
		3	0,04	0,05	1,00	-0,08	0,17
	3	1	0,01	0,03	1,00	-0,06	0,08
		2	-0,04	0,05	1,00	-0,17	0,08
Sham	1	2	-0,01	0,05	1,00	-0,13	0,11
		3	0,00	0,03	1,00	-0,07	0,07
	2	1	0,01	0,05	1,00	-0,11	0,13
		3	0,01	0,05	1,00	-0,12	0,13
	3	1	0,00	0,03	1,00	-0,07	0,07
		2	-0,01	0,05	1,00	-0,13	0,12

LEGEND: MD - Mean difference SD - Standard Deviation, CI - confidence interval

Supplementary Table 9. The effects of experimental manipulation on MES: main effect (interaction between assessment and group), effect of assessment and effect of group.

	SS	*df*	Mean Square	*F*	*p*	η^2
**Total sample (*N*=91)**						
Assessment	3630,51	1,15	3160,97	8,73	0,003*	0,09
Assessment * Group	6022,78	2,30	1505,69	7,24	<,001*	0,141
Error (Phase)	36608,94	101,07	362,21			
Group	6077,83	2,00	3038,92	11,95	<.001*	0,214

LEGEND: SS - Type III Sum of Squares, df - degrees of freedom, η^2 - partial eta squared

Supplementary Table 9a. The effects of experimental manipulation on MES: main effect (interaction between assessment and group), effect of assessment and effect of group with covariants (WDI and GF)

	SS	*df*	Mean Square	*F*	*p*	η^2
**Total sample (*N*=91)**						
Assessment	65,13	1,16	56,30	0,16	0,73	0,002
Assessment * GF	438,08	1,16	378,67	1,09	0,31	0,012
Assessment * WDI	1273,90	1,16	1101,15	3,16	0,07	0,035
Assessment * Group	6002,88	2,31	2594,42	7,43	<,001*	0,147
Error (Phase)	34721,51	99,49	348,99			
Group	6023,82	2,00	3011,91	12,23	<.001*	0,221

LEGEND: SS - Type III Sum of Squares, df - degrees of freedom, η^2 - partial eta squared, WDI – weight discrepancy index, GF – general fear

Supplementary Table 9b. The effects of experimental manipulation on MES: results of pairwise comparisons (between groups in each assessment)

Assessment	Group	Group	MD	SD	p	95% CI	
						Lower	Upper
1	Injection	Control	0	0	.	0	0
		Sham	0	0	.	0	0
	Control	Injection	0	0	.	0	0
		Sham	0	0	.	0	0
	Sham	Injection	0	0	.	0	0
		Control	0	0	.	0	0
2	**Injection**	**Control**	23,66	6,53	0,00*	7,70	39,61
		**Sham**	25,39	6,47	<,001*	9,61	41,18
	**Control**	**Injection**	-23,66	6,53	0,00*	-39,61	-7,70
		Sham	1,74	6,43	1,00	-13,96	17,43
	**Sham**	**Injection**	-25,39	6,47	<,001*	-41,18	-9,61
		Control	-1,74	6,43	1,00	-17,43	13,96
3	**Injection**	**Control**	5,71	2,07	0,02*	0,65	10,78
		**Sham**	7,38	2,05	0,00*	2,37	12,39
	**Control**	**Injection**	-5,71	2,07	0,02*	-10,78	-0,65
		Sham	1,67	2,04	1,00	-3,32	6,65
	**Sham**	**Injection**	-7,38	2,05	0,00*	-12,39	-2,37
		Control	-1,67	2,04	1,00	-6,65	3,32

LEGEND: MD - Mean difference SD - Standard Deviation, CI - confidence interval

Supplementary Table 9c. The effects of experimental manipulation on MES: results of pairwise comparisons (between assessments in each group)

Assessment	Group	Group	MD	SD	p	95% CI	
						**Lower**	**Upper**
Injection	**1**	**2**	-24,70	4,51	<,001*	-35,72	-13,68
		**3**	-5,80	1,43	<,001*	-9,30	-2,30
	**2**	**1**	24,70	4,51	<,001*	13,68	35,72
		**3**	18,80	4,40	<,001*	8,16	29,64
	**3**	**1**	5,80*	1,43	<,001*	2,30	9,30
		**2**	-18,90	4,40	<,001*	-29,64	-8,16
Control	1	2	-1,04	4,55	1,00	-12,14	10,06
		3	-0,09	1,44	1,00	-3,61	3,44
	2	1	1,04	4,55	1,00	-10,06	12,14
		3	0,95	4,43	1,00	-9,87	11,78
	3	1	0,09	1,44	1,00	-3,44	3,61
		2	-0,95	4,43	1,00	-11,78	9,87
Sham	1	2	0,70	4,51	1,00	-10,32	11,72
		3	1,58	1,43	0,82	-1,92	5,08
	2	1	-0,70	4,51	1,00	-11,72	10,32
		3	0,88	4,40	1,00	-9,86	11,63
	3	1	-1,58	1,43	0,82	-5,08	1,92
		2	-0,88	4,40	1,00	-11,63	9,86

LEGEND: MD - Mean difference SD - Standard Deviation, CI - confidence interval

Supplementary Table 10. The effects of experimental manipulation on DR_R: main effect (interaction between assessment and group), effect of assessment and effect of group.

	SS	*df*	Mean Square	*F*	*p*	η^2
**Total sample (*N*=91)**						
Assessment	184,08	1,96	93,99	2,22	0,116	0,053
Assessment * Group	557,29	3,92	142,27	3,37	0,014*	0,144
Error (Phase)	3312,24	78,34	42,28			
Group	1290,51	2,00	645,25	2,67	0,081	0,118

LEGEND: SS - Type III Sum of Squares, df - degrees of freedom, η^2 - partial eta squared

Supplementary Table 10a. The effects of experimental manipulation on DR_R: main effect (interaction between assessment and group), effect of assessment and effect of group with covariants (WDI and GF)

	SS	*df*	Mean Square	*F*	*p*	η^2
**Total sample (*N*=91)**						
Assessment	30,49	1,94	15,71	0,37	0,686	0,01
Assessment * GF	119,69	1,94	61,66	1,45	0,241	0,037
Assessment * WDI	46,51	1,94	23,96	0,56	0,567	0,015
Assessment * Group	415,20	3,88	106,95	2,51	0,05*	0,117
Error (Phase)	3137,55	73,76	42,54	3137,55		
Group	1850,86	2,00	925,43	4,00	0,03*	0,174

LEGEND: SS - Type III Sum of Squares, df - degrees of freedom, η^2 - partial eta squared, WDI – weight discrepancy index, GF – general fear

Supplementary Table 10b. The effects of experimental manipulation on DR_R: results of pairwise comparisons (between groups in each assessment)

Assessment	Group	Group	MD	SD	p	95% CI	
						Lower	Upper
1	Injection	Control	8,59	4,40	0,18	-2,43	19,61
		Sham	2,21	4,30	1,00	-8,57	12,98
	Control	Injection	-8,59	4,40	0,18	-19,61	2,43
		Sham	-6,38	4,68	0,54	-18,10	5,33
	Sham	Injection	-2,21	4,30	1,00	-12,98	8,57
		Control	6,38	4,68	0,54	-5,33	18,10
2	Injection	Control	15,85	3,57	<,001*	6,90	24,80
		Sham	8,25	3,49	0,07	-0,50	17,00
	Control	Injection	-15,85	3,57	<,001*	-24,80	-6,90
		Sham	-7,61	3,80	0,16	-17,12	1,91
	Sham	Injection	-8,25	3,49	0,07	-17,00	0,50
		Control	7,61	3,80	0,16	-1,91	17,12
3	Injection	Control	5,18	4,19	0,67	-5,31	15,66
		Sham	1,75	4,09	1,00	-8,50	11,99
	Control	Injection	-5,18	4,19	0,67	-15,66	5,31
		Sham	-3,43	4,45	1,00	-14,58	7,71
	Sham	Injection	-1,75	4,09	1,00	-11,99	8,50
		Control	3,43	4,45	1,00	-7,71	14,58

LEGEND: MD - Mean difference SD - Standard Deviation, CI - confidence interval

Supplementary Table 10c. The effects of experimental manipulation on DR_R: results of pairwise comparisons (between assessments in each group)

Assessment	Group	Group	MD	SD	p	95% CI	
						Lower	Upper
Injection	1	2	-4,96	2,44	0,15	-11,07	1,15
		3	3,51	2,33	0,42	-2,32	9,34
	2	1	4,96	2,44	0,15	-1,15	11,07
		3	8,47	2,10	<,001*	3,22	13,72
	3	1	-3,51	2,33	0,42	-9,34	2,32
		2	-8,47	2,10	<,001*	-13,72	-3,22
Control	1	2	2,30	2,77	1,00	-4,65	9,25
		3	0,10	2,65	1,00	-6,54	6,73
	2	1	-2,30	2,77	1,00	-9,25	4,65
		3	-2,21	2,38	1,00	-8,17	3,77
	3	1	-0,10	2,65	1,00	-6,73	6,54
		2	2,21	2,38	1,00	-3,77	8,17
Sham	1	2	1,08	2,87	1,00	-6,10	8,26
		3	3,05	2,74	0,82	-3,81	9,90
	2	1	-1,08	2,87	1,00	-8,26	6,10
		3	1,97	2,46	1,00	-4,20	8,14
	3	1	-3,05	2,74	0,82	-9,90	3,81
		2	-1,97	2,46	1,00	-8,14	4,20

LEGEND: MD - Mean difference SD - Standard Deviation, CI - confidence interval

Supplementary Table 11. The effects of experimental manipulation on DR_L: main effect (interaction between assessment and group), effect of assessment and effect of group.

	SS	*df*	Mean Square	*F*	*p*	η^2
			**Total sample (*N*=91)**			
Assessment	10,29	1,78	5,79	0,11	0,875	0,002
Assessment * Group	101,45	3,55	28,57	0,54	0,687	0,023
Error (Phase)	4243,65	79,89	53,12			
Group	312,81	2,00	156,41	0,62	0,54	0,027

LEGEND: SS - Type III Sum of Squares, df - degrees of freedom, η^2 - partial eta squared

Supplementary Table 11a. The effects of experimental manipulation on DR_L: main effect (interaction between assessment and group), effect of assessment and effect of group with covariants (WDI and GF)

	SS	*df*	Mean Square	*F*	*p*	η^2
			**Total sample (*N*=91)**			
Assessment	104,30	1,75	59,76	1,11	0,329	0,025
Assessment * GF	106,70	1,75	61,13	1,13	0,321	0,026
Assessment * WDI	95,80	1,75	54,89	1,02	0,357	0,023
Assessment * Group	124,31	3,49	35,61	0,66	0,601	0,03
Error (Phase)	4047,12	75,05	53,93			
Group	456,08	2,00	228,04	0,96	0,39	0,043

LEGEND: SS - Type III Sum of Squares, df - degrees of freedom, η^2 - partial eta squared, WDI – weight discrepancy index, GF – general fear

Supplementary Table 11b. The effects of experimental manipulation on DR_L: results of pairwise comparisons (between groups in each assessment)

Assessment	Group	Group	MD	SD	p	95% CI	
						Lower	Upper
1	Injection	Control	2,69	3,95	1,00	-7,15	12,53
		Sham	1,34	3,94	1,00	-8,48	11,15
	Control	Injection	-2,69	3,95	1,00	-12,53	7,15
		Sham	-1,35	3,69	1,00	-10,55	7,85
	Sham	Injection	-1,34	3,94	1,00	-11,15	8,48
		Control	1,35	3,69	1,00	-7,85	10,55
2	Injection	Control	4,62	4,00	0,76	-5,33	14,57
		Sham	1,64	3,99	1,00	-8,29	11,57
	Control	Injection	-4,62	4,00	0,76	-14,57	5,33
		Sham	-2,98	3,74	1,00	-12,29	6,32
	Sham	Injection	-1,64	3,99	1,00	-11,57	8,29
		Control	2,98	3,74	1,00	-6,32	12,29
3	Injection	Control	6,37	3,81	0,31	-3,12	15,85
		Sham	6,21	3,80	0,33	-3,25	15,68
	Control	Injection	-6,37	3,81	0,31	-15,85	3,12
		Sham	-0,15	3,56	1,00	-9,02	8,71
	Sham	Injection	-6,21	3,80	0,33	-15,68	3,25
		Control	0,15	3,56	1,00	-8,71	9,02

LEGEND: MD - Mean difference SD - Standard Deviation, CI - confidence interval

Supplementary Table 11c. The effects of experimental manipulation on DR_L: results of pairwise comparisons (between assessments in each group)

Assessment	Group	Group	MD	SD	p	95% CI	
						Lower	Upper
Injection	1	2	-0,05	2,64	1,00	-6,63	6,53
		3	-2,58	3,08	1,00	-10,25	5,09
	2	1	0,05	2,64	1,00	-6,53	6,63
		3	-2,53	2,19	0,76	-7,99	2,93
	3	1	2,58	3,08	1,00	-5,09	10,25
		2	2,53	2,19	0,76	-2,93	7,99
Control	1	2	1,89	2,42	1,00	-4,14	7,91
		3	1,10	2,82	1,00	-5,93	8,13
	2	1	-1,89	2,42	1,00	-7,91	4,14
		3	-0,79	2,01	1,00	-5,78	4,21
	3	1	-1,10	2,82	1,00	-8,13	5,93
		2	0,79	2,01	1,00	-4,21	5,78
Sham	1	2	0,25	2,32	1,00	-5,52	6,02
		3	2,30	2,70	1,00	-4,43	9,02
	2	1	-0,25	2,32	1,00	-6,02	5,52
		3	2,04	1,92	0,88	-2,74	6,83
	3	1	-2,30	2,70	1,00	-9,02	4,43
		2	-2,04	1,92	0,88	-6,83	2,74

LEGEND: MD - Mean difference SD - Standard Deviation, CI - confidence interval

Supplementary Table 12. The effects of experimental manipulation on BES: main effect (interaction between assessment and group), effect of assessment and effect of group.

	SS	*df*	Mean Square	*F*	*p*	η^2
**Total sample (*N*=91)**						
Assessment	193,99	1,75	111,12	5,79	,005*	0,062
Assessment * Group	171,996	3,49	49,26	2,57	,048*	0,055
Error (Phase)	2946,05	153,62	19,18			
Group	1107,69	2	553,84	0,48	,617	0,011

LEGEND: SS - Type III Sum of Squares, df - degrees of freedom, η^2 - partial eta squared

Supplementary Table 12a. The effects of experimental manipulation on BES: main effect (interaction between assessment and group), effect of assessment and effect of group with covariants (WDI and GF)

	SS	*df*	Mean Square	*F*	*p*	η^2
**Total sample (*N*=91)**						
Assessment	56,41	1,75	32,17	1,73	0,185	0,02
Assessment * GF	31,35	1,75	17,88	0,96	0,376	0,011
Assessment * WDI	102,53	1,75	58,48	3,14	0,053	0,035
Assessment * Group	175,18	3,51	49,95	2,68	0,041*	0,059
Error (Phase)	2811,56	150,79	18,64			
Group	1101,50	2	550,75	0,56	,572	0,013

LEGEND: SS - Type III Sum of Squares, df - degrees of freedom, η^2 - partial eta squared, WDI – weight discrepancy index, GF – general fear

Supplementary Table 12b. The effects of experimental manipulation on BES: results of pairwise comparisons (between groups in each assessment)

Assessment	Group	Group	MD	SD	p	95% CI	
						Lower	Upper
1	Injection	Control	4,85	4,89	0,97	-7,10	16,80
		Sham	0,30	4,84	1,00	-11,53	12,12
	Control	Injection	-4,85	4,89	0,97	-16,80	7,10
		Sham	-4,56	4,81	1,00	-16,31	7,20
	Sham	Injection	-0,30	4,84	1,00	-12,12	11,53
		Control	4,56	4,81	1,00	-7,20	16,31
2	Injection	Control	0,80	4,99	1,00	-11,38	12,97
		Sham	-3,68	4,94	1,00	-15,73	8,37
	Control	Injection	-0,80	4,99	1,00	-12,97	11,38
		Sham	-4,48	4,91	1,00	-16,46	7,50
	Sham	Injection	3,68	4,94	1,00	-8,37	15,73
		Control	4,48	4,91	1,00	-7,50	16,46
3	Injection	Control	4,28	4,95	1,00	-7,80	16,36
		Sham	-1,67	4,90	1,00	-13,63	10,28
	Control	Injection	-4,28	4,95	1,00	-16,36	7,80
		Sham	-5,95	4,87	0,68	-17,83	5,94
	Sham	Injection	1,67	4,90	1,00	-10,28	13,63
		Control	5,95	4,87	0,68	-5,94	17,83

LEGEND: MD - Mean difference SD - Standard Deviation, CI - confidence interval

Supplementary Table 12c. The effects of experimental manipulation on BES: results of pairwise comparisons (between assessments in each group)

Assessment	Group	Group	MD	SD	p	95% CI	
						Lower	Upper
Injection	1	2	4,64	1,18	<,001*	1,75	7,52
		3	2,38	1,13	0,12	-0,38	5,13
	2	1	-4,64	1,18	<,001*	-7,52	-1,75
		3	-2,26	0,84	0,03*	-4,32	-0,20
	3	1	-2,38	1,13	0,12	-5,13	0,38
		2	2,26	0,84	0,03*	0,20	4,32
Control	1	2	0,58	1,19	1,00	-2,33	3,49
		3	1,80	1,14	0,35	-0,98	4,58
	2	1	-0,58	1,19	1,00	-3,49	2,33
		3	1,22	0,85	0,46	-0,85	3,29
	3	1	-1,80	1,14	0,35	-4,58	0,98
		2	-1,22	0,85	0,46	-3,29	0,85
Sham	1	2	0,66	1,18	1,00	-2,23	3,55
		3	0,41	1,13	1,00	-2,35	3,17
	2	1	-0,66	1,18	1,00	-3,55	2,23
		3	-0,25	0,84	1,00	-2,31	1,81
	3	1	-0,41	1,13	1,00	-3,17	2,35
		2	0,25	0,84	1,00	-1,81	2,31

LEGEND: MD - Mean difference SD - Standard Deviation, CI - confidence interval

Supplementary Table 13. Variables across the three groups (injection, sham, control)

		Injection group		Sham-injection group		Control group	
	Assessment	M	SD	M	SD	M	SD
Primary outcome							
FreBAQ_PL	I	4,00	3,86	4,27	4,29	4,47	4,19
FreBAQ_PL	II	8,94	6,69	3,83	4,67	3,87	4,44
FreBAQ_PL	III	4,52	4,94	3,40	4,69	3,47	3,58
Secondary outcome							
APD	I	0,00%	0,00%	0,00%	0,00%	0,00%	0,00%
APD	II	0,55%	0,42%	0,01%	0,05%	0,04%	0,17%
APD	III	0,08%	0,25%	0,00%	0,01%	0,01%	0,06%
PT	I	4,85	3,13	4,97	3,20	4,80	3,08
PT	II	3,37	2,10	3,78	2,82	3,60	2,12
PT	III	3,48	1,86	3,52	2,72	3,53	2,01
DT	I	0,71	0,34	0,68	0,33	0,68	0,40
DT	II	0,73	0,36	0,60	0,31	0,65	0,35
DT	III	0,66	0,27	0,58	0,30	0,63	0,39
TPE_V	I	88,48	30,33	87,93	19,36	91,53	24,01
TPE_V	II	90,81	27,38	97,10	22,24	106,12	22,06
TPE_V	III	93,29	28,74	101,17	21,30	104,83	21,56
TPE_H	I	72,23	25,21	78,10	22,39	74,48	24,46
TPE_H	II	81,37	30,55	87,02	23,78	83,22	25,58
TPE_H	III	81,03	26,22	87,23	27,64	81,30	25,52
FE	I	-1,76	1,78	-1,53	1,48	-2,03	1,58
FE	II	-1,50	1,70	-1,33	1,56	-1,87	1,52
FE	III	-1,37	1,75	-1,23	1,59	-1,90	1,53
MES	I	0,00	0,00	0,00	0,00	0,00	0,00
MES	II	23,96	39,06	1,10	16,68	0,00	0,00
MES	III	5,74	11,55	-1,44	6,25	0,00	0,00
BD_L	I	129,85	9,43	130,60	9,57	128,47	12,90
BD_L	II	130,58	14,05	130,09	6,10	126,28	11,33
BD_L	III	132,21	13,98	128,72	7,28	127,10	10,80
BD_R	I	134,83	11,59	134,07	8,86	127,75	12,05
BD_R	II	139,41	9,27	132,88	7,72	126,00	10,50
BD_R	III	130,93	10,10	129,94	11,44	129,06	10,86
BES	I	129,48	18,67	125,23	21,13	123,40	18,00
BES	II	125,23	20,82	124,10	20,96	122,90	18,71
BES	III	127,65	19,99	124,50	21,00	121,37	18,59

LEGEND: N – number of participants, M – mean, SD – standard deviation, F – statistic, $\eta 2$ – partial eta squared, FreBAQ_PL – Fremantle Back Awareness Questionnaire, PT – pain threshold, DT – detection threshold, TPE – two point estimation, V – vertical, H – horizontal, MES – Magnitude estimation scale, BD – back drawings, L – left side of stimulation, R – right side of stimulation, BES – body esteem scale
